# Role of inflammasomes in multiple sclerosis and their potential as therapeutic targets

**DOI:** 10.1186/s12974-020-01944-9

**Published:** 2020-09-02

**Authors:** Vaidya Govindarajan, Juan Pablo de Rivero Vaccari, Robert W. Keane

**Affiliations:** 1 Department of Physiology and Biophysics, University of Miami Miller School of Medicine, 1600 NW 10th Ave RMSB 5058, Miami, FL 33136 USA; 2 Department of Neurological Surgery and The Miami Project to Cure Paralysis, University of Miami Miller School of Medicine, Miami, FL 33136 USA

**Keywords:** Inflammasome, multiple sclerosis, EAE, caspase-1, IL-1β

## Abstract

Multiple sclerosis (MS) is a demyelinating disease of the central nervous system (CNS), and it remains the most common immune-mediated disorder affecting the CNS. While the cause of MS is unclear, the underlying pathomechanisms are thought to be either destruction by autoimmune T cells or dysfunction of myelin-producing cells. Recent advances have indicated that inflammasomes contribute the etiology of MS. Inflammasomes are multiprotein complexes of the innate immune response involved in the processing of caspase-1, the activation of pro-inflammatory cytokines interleukin (IL)-1β and IL-18 as well as the cell death-mediated mechanism of pyroptosis and the activation of the adaptive immune response. Here we review the literature to date on the role of different inflammasome signaling pathways in the pathogenesis of MS and how these pathways may be targeted to reduce deleterious inflammatory processes and improve outcomes in this patient population.

## Background/introduction

**Fig. 1:**
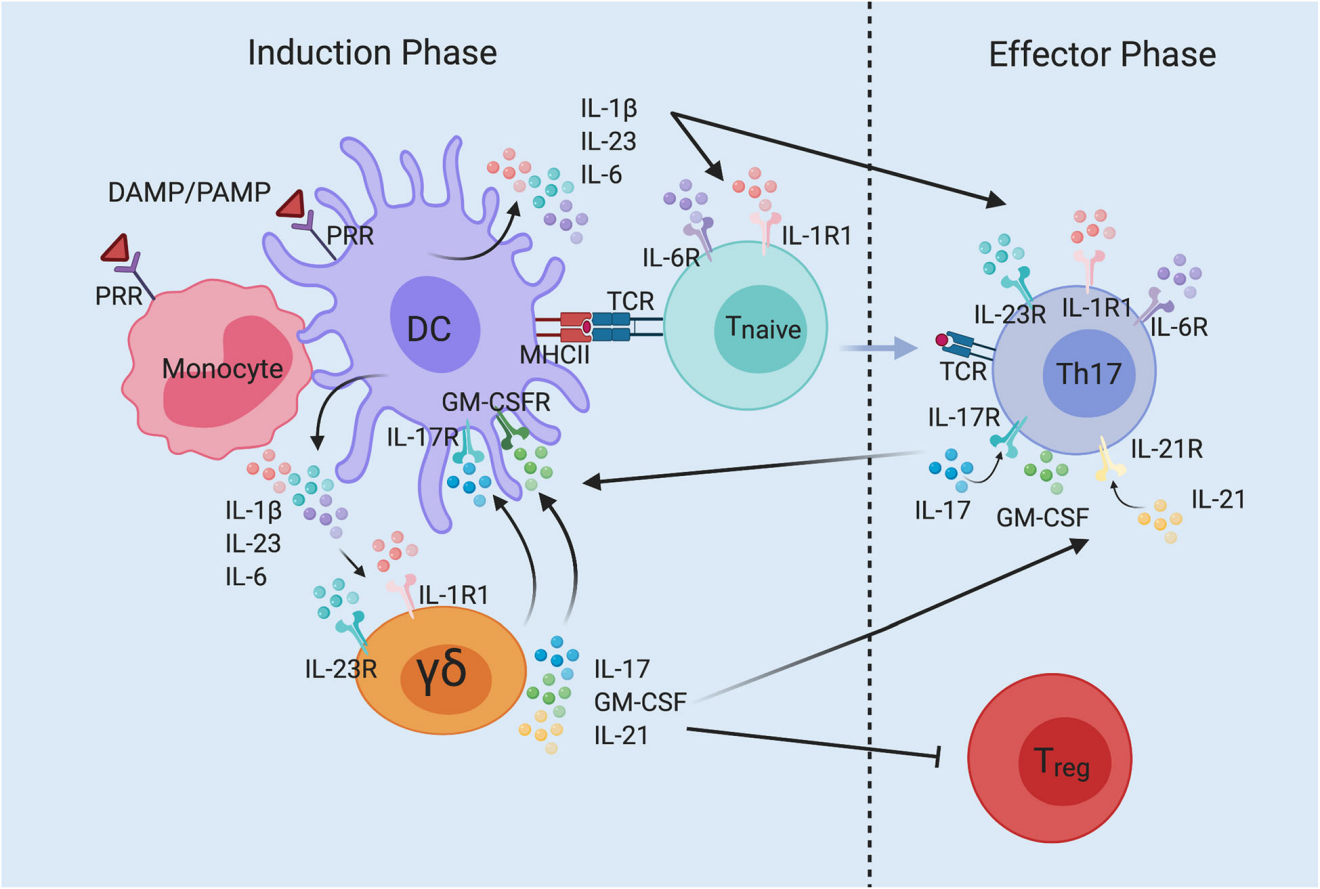
Induction and interactions of γδ T cell and Th17 cell signaling pathways in MS and EAE. In the induction phase, pathogen-associated molecular patterns (PAMP) from commensal bacteria and danger-associated molecular patterns (DAMP) from dead/dying cells bind to pattern recognition receptors (PRR) on monocytes and dendritic cells (DC). Activated DC and monocytes secrete interleukin (IL)-β, IL-23, and IL-6. IL-β and IL-23 activate γδ T cells leading to IL-17, IL-21, and granulocyte-macrophage colony stimulating factor (GM-CSF) production. Activated DC present antigenic peptide by MHC class II molecules to T cell receptors (TCR) on T_naive_ (CD4) T cells. Antigenic peptide presentation along with co-stimulatory signals from DC (IL1-β, IL-6 and IL-23) activates Th17 cells that respond and secrete IL-17, GM-CSF, and IL-21. These cytokines are also released from activated γδ T cells and jointly combine to initiate pro-inflammatory feedback loops that augment the production of IL-1β, IL-6, and IL-23 by antigen presenting cells (APC), leading to enhanced Th17 responses and continued γδ T cell activation of the effector phase. IL-17 and IL-21 act in an autocrine manner to enhance IL-17 production by Th17 T cells in the development of EAE. Lastly, activated γδ T cells suppress T regulatory (T_reg_) responses, promoting the pro-inflammatory profile by effector T cells. Adapted from McGinley et al. [1]

**Fig. 2:**
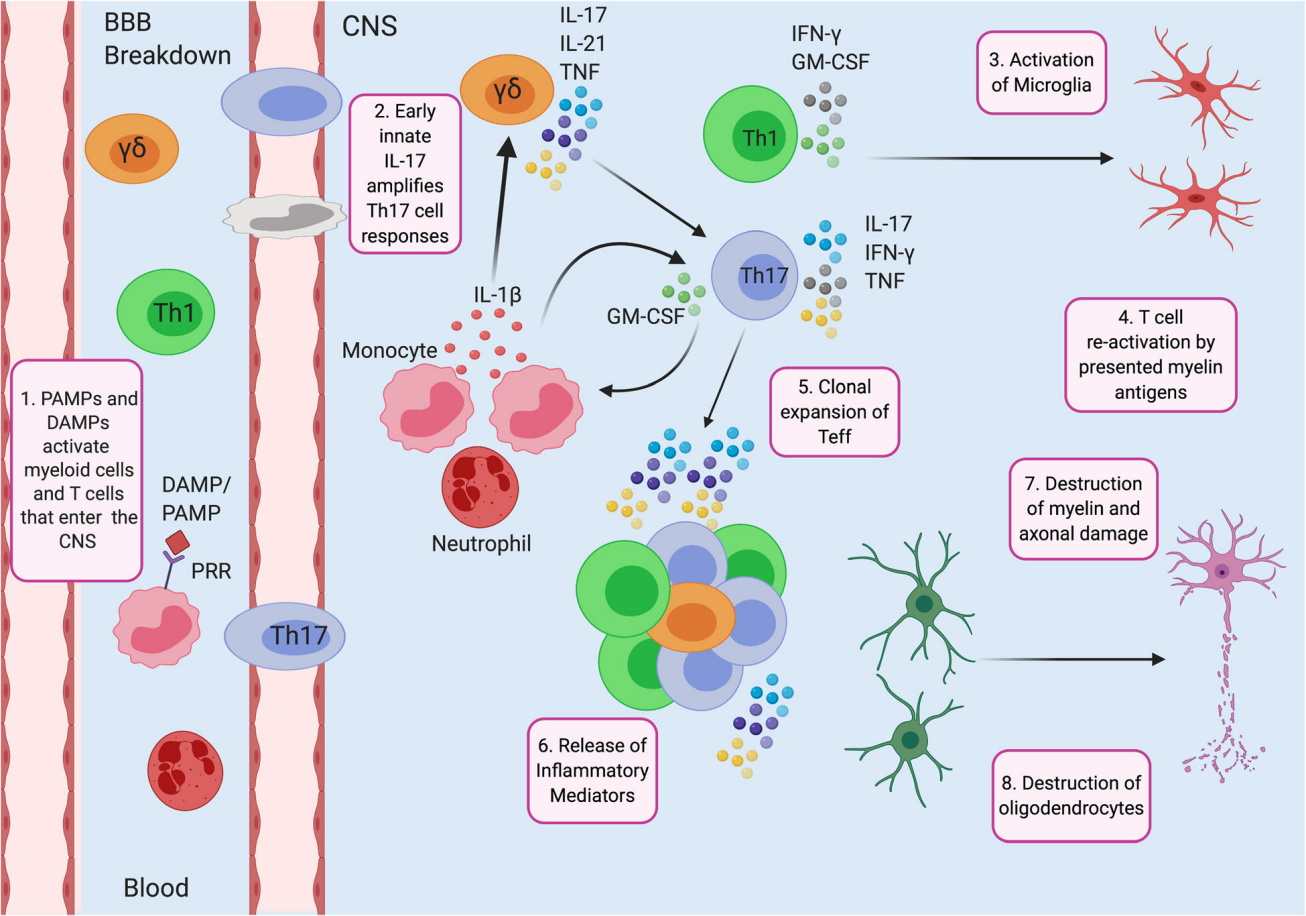
Key pathological processes within blood and central nervous system (CNS) during EAE and MS. (1) Danger associate molecular patterns (DAMP) and Pathogen-Associated molecular patterns (PAMP) released into the peripheral blood activate inflammatory myeloid and T cells that migrate into the CNS after blood-brain barrier (BBB) breakdown. (2) Infiltrating monocytes and neutrophils release IL-1β that activates γδ T cells to secrete IL-17, IL-21 and blood-brain barrier (TNF), which amplifies Th17 responses. In addition, activation of inflammatory myeloid cells releases granulocyte-macrophage colony stimulating factor (GM-CSF) that amplifies Th17 cell activation (3) IFN-γ and GM-CSF released from Th1 cells, and IL-17, interferon (IFN)-γ and tumor necrosis factor (TNF) released from activated Th17 cells activate microglia. (4) T cell re-activation by presented myelin antigens. (5) Clonal expansion of T effector (Teff) cells. (6) Activation of infiltrating and resident cells results in the release of inflammatory mediators by Th1 and Th17 cells (IL-17, GM-CSF, IFN-γ, TNF) and production of reactive oxygen and nitrogen species, cytotoxic products and proteases that promote the destruction of myelin around axons (7) and oligodendrocytes (8), which are responsible for axonal remyelination, resulting in a toxic inflammatory response that leads to neuronal death and neurological deficits. Adapted from McGinley et al. [1]

Multiple sclerosis (MS) and the animal model of MS, experimental autoimmune encephalomyelitis (EAE), are autoimmune demyelinating diseases of the central nervous system (CNS) characterized by the development of myelin-reactive CD4 T cells, Th1, Th17, T_reg_, γδ T cells and B cells (Fig. 1). Investigations in the EAE model have identified key immune cells mediating inflammation in the CNS. In the induction phase of EAE, inflammation is initiated by the binding of pathogen-associated molecular patterns (PAMP) or danger-associated molecular patterns (DAMP) to pattern recognition receptors (PRR) on innate immune cells, including inflammatory dendritic cells and monocytes (Fig. 1). PRR receptor binding results in production of interleukin (IL)-1, IL-6, IL-12, IL-18, and IL-23 that promote the induction and expansion of Th1 and Th17 cells (Fig. 1) [1]. This process is further amplified by IL-1β- and IL-23-activated γδ T cells that secrete IL-17 and IL-21 that act in a feedback loop to enhance the Th17 response as part of the effector phase. Following breakdown of the blood-brain barrier (BBB), activation of Th1, Th17 and γδ T cells enter the brain and spinal cord. In addition, resident microglia of the CNS, infiltrating monocytes and neutrophils secrete IL-1β and IL-23 to further activate and expand these cells (Fig. 2). Infiltrating γδ T cells, Th1 and Th17 secrete the inflammatory cytokines IL-17, granulocyte and macrophage colony stimulation factor (GM-CSF), interferon gamma (IFN-γ) and tumor necrosis factor (TNF) that activate microglia and oligodendroglia inducing the production of inflammatory mediators such as IL-8 that recruits neutrophils and promotes the production of matrix metalloproteinases and chemokines. Together, these inflammatory responses lead to myelin and axonal damage resulting in neurodegeneration (Fig. 2).

The role of B cells in MS pathogenesis has been studied for decades, with some of the earliest work in MS revolving around the increased abundance of immunoglobulins in the cerebrospinal fluid (CSF). Specifically, immunoglobulin G (IgG) oligoclonal bands have been a long-standing biomarker of MS, though immunoglobulin M (IgM) oligoclonal bands have also demonstrated elevation in MS patients and predict a more aggressive disease course [2].

With respect to B cell autoreactivity, it has been previously demonstrated that T_reg_ dysfunction leads to inadequate suppression of autoreactive B cells [3]. Specifically, forkhead box protein 3 (*FOXP3*) deficiencies were associated with increased accumulation of autoreactive B cells, indicating that T_reg_ cells are critical in suppressing B cell autoreactivity [3]. A later study by the same group linked deficiencies in the peripheral B cell tolerance checkpoint (i.e., secondary lymphoid organs) to MS pathogenesis, likely attributable to T_reg_ dysfunction [3].

Despite these correlations, the exact role of B cells in MS pathogenesis remains unclear but may involve the upregulation of pro-inflammatory cytokines [4]. For example, upregulation of IL-6 by B cells activates Th17 responses in MS patients [4]. B cells may also further exacerbate MS pathogenesis by inducing auto-proliferation of Th1 cells, and this population of Th1 cells is enriched with “brain-homing” Th1 cells [5]. Thus, there appears to be reciprocal mechanisms in T and B cells that are responsible for the inflammatory cascade underlying MS pathogenesis.

The inflammatory process in MS manifests as CNS plaques and areas of focal demyelination within the white matter of the brain, spinal cord, and optic nerve. MS plaques are frequently associated with blood vessels and periventricular regions with the presence of inflammatory infiltrates containing activated macrophages, microglia, and T cells [6]. The initial symptoms usually manifest in the second and third decades of life [7]. MS symptoms coincide with the occurrence of focal plaques, with the most common presentations being optic neuritis (a product of demyelination of the optic nerve) and transverse myelitis (sensory symptoms produced by demyelination of the dorsolateral spinal cord) [8, 9]. Other symptoms include ataxia (due to cerebellar lesions), cognitive impairment, and more rarely, aphasia, encephalopathy, and increased intracranial pressure. These latter symptoms are typically caused by large hemispheric lesions [8].

The progression of MS is highly variable and may present as any of three forms: relapsing-remitting (RRMS, focal symptoms present and then spontaneously resolve), primary progressive (PPMS, relentless progression of focal lesions and deficits that do not resolve), and secondary progressive (SPMS, alteration from RRMS to PPMS). While PPMS is found in 15% of patients, upwards of 50% of RRMS go on to develop SPMS due to lack of treatment [10]. Thus, it is important to gain a clear understanding of pathomechanisms involved in MS in order to develop better therapies for the treatment of this devastating autoimmune disease. Here, we review the literature on the inflammasome signaling pathways involved the pathology of MS, and the therapeutic potential of modulation of inflammasome signaling to improve outcomes in this patient population.

## Inflammasomes: overview

**Fig. 3:**
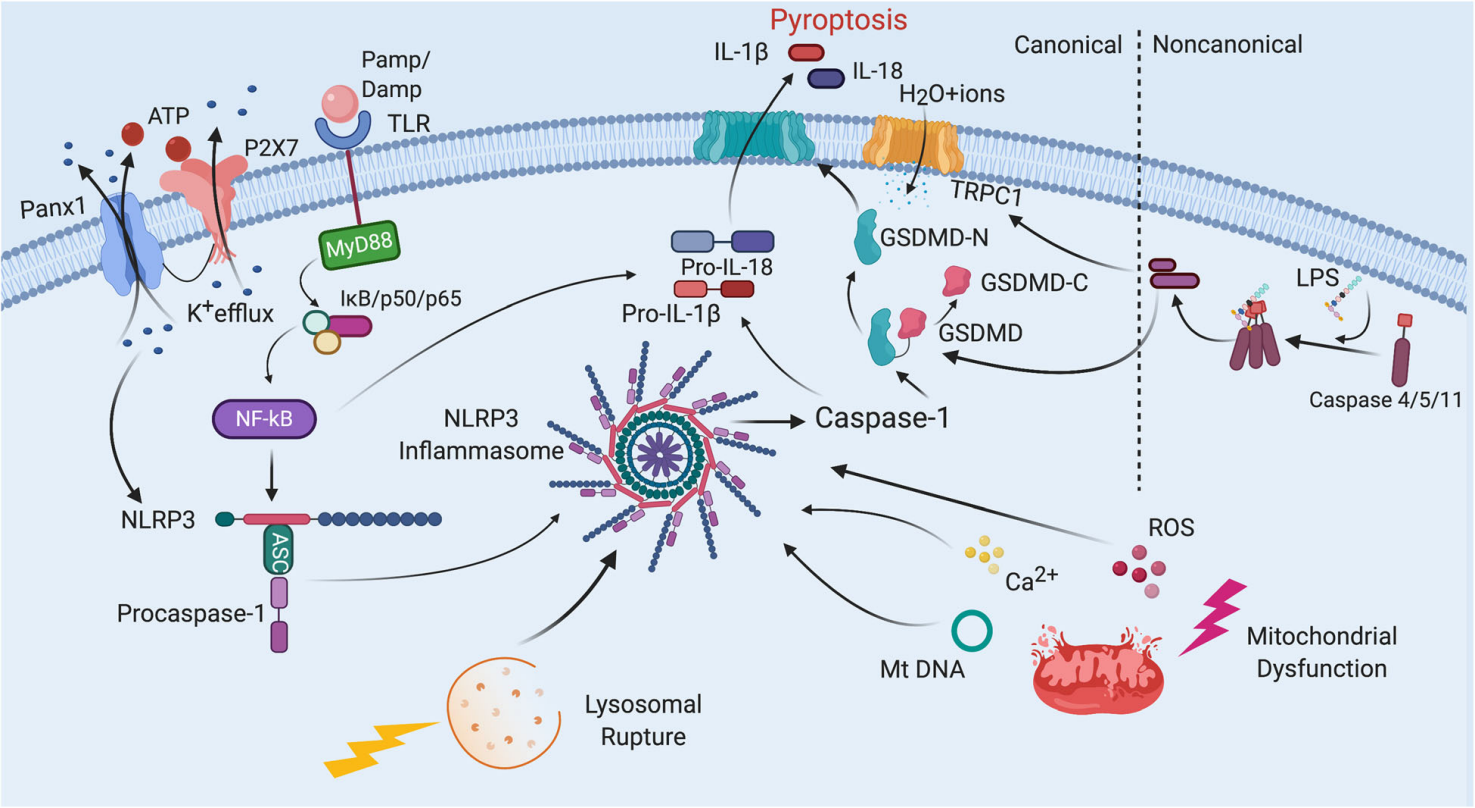
Mechanism of action of the NLRP3 inflammasome. In the canonical pathway, the priming step involves the recognition of pathogen-associated molecular pattern (PAMP) or a danger-associated molecular pattern (DAMP) by a pattern recognition receptor (PRR) or toll-like receptor (TLR), which recruits the adaptor protein myeloid differentiation primary response 88 (MYD88) into the receptor complex leading to phosphorylation of the inhibitor of nuclear factor-kB (NFκB) IκB through interaction with the p50 and p65 transcription factors. Activation of the NF-κB pathway causes the synthesis of pro-IL-1-β or pro-IL-18. NLRP3 is activated by lysosomal-mediated cathepsin B via lysosome rupture, reactive oxygen species (ROS), oxidized mitochondrial (Mt) DNA, altered Ca2^+^ concentration via mitochondrial dysfunction, and adenosine triphosphate (ATP) efflux and potassium (K^+^) via pannexin1 (Panx1) channels. High extracellular ATP acts as a DAMP and binds to the purinergic receptor, P2X7 causing additional K^+^ efflux. Activation of NLRP3 involves oligomerization that results in activation of caspase-1 that converts pro-IL1β and pro-IL-18 into their mature forms. In addition, active caspase-1 cleaves Gasdermin D (GSDMD) into an N-terminus domain (GSDMD-N) and the autoinhibitory C-terminus domain (GSDMD-C). GSDMD-N binds to acidic phospholipids in the inner leaflet of the plasma membrane and oligomerizes to form pores that disrupt plasma membrane integrity, enabling release of IL-1β and IL-18 and inducing pyroptosis. The non-canonical pathway is activated by gram-negative bacteria or lipopolysaccharide (LPS) to activate caspase 4/5 in humans or caspase-11 in rodents that cleave GSDMD. The transient receptor protein channel 1 (TRPC1), a non-selective ion channel is also a substrate for caspase-11

A previous study [7] provides evidence that inflammasome activation plays a critical role in the autoimmune and pro-inflammatory responses in MS. Inflammasomes are large protein complexes that play a critical role in the innate immune response against DAMPs released from dead and dying cells and PAMPs from bacteria or virus. Inflammasome assembly results in caspase-1 activation, which in turn leads to the production and secretion of mature IL-1β and IL-18 [11]. Excessive caspase-1 induction leads to a programmed cell death process termed pyroptosis characterized by rapid induction of an inflammatory response leading to cell lysis [12] (Fig. 3).

Inflammasomes are composed of three components: an inflammasome sensory protein such as a NOD-like receptor (NLR), the adaptor protein apoptosis-associated speck-like protein containing a caspase-recruitment domain (ASC), and caspase-1 [11]. The toll-like receptor (TLR) and NLR proteins are a families of PRR necessary for the recognition of PAMP and DAMP, markers of pathogens and cell damage, respectively [13]. PRR receptor binding activates a priming of the inflammasome (often referred to as signal 1), leading to activation of nuclear factor-κB (NF-κB). The ASC protein is particularly critical, as it serves to link the sensory protein (NLR) to caspase-1 [14] (Fig. 3).

In unstimulated cells, ASC is found in mitochondria, nucleus, and cytosol. Inflammasome induction relocates ASC into the cytosol to facilitate interaction with the sensor (NLR, AIM2 like receptor (ALR), pryin) [15]. This change in cellular distribution of ASC is influenced by mitochondrial microtubule modulations and homeostasis, indicating that cytoskeletal components play a role in inflammasome regulation [16].

In the canonical pathway, inflammasome activation results in production of mature IL-1β and IL-18 in response to infection or tissue damage or a variety of metabolic disturbances, including lysosomal disruption, mitochondrial dysfunction, release of mitochondrial DNA (mt DNA), reactive oxygen species (ROS), and high intracellular calcium (Ca^2+^). Binding of a PAMP or DAMP to the NLR portion of the inflammasome leads to oligomerization of NLR proteins *via* homotypic pyrin (PYD) or caspase-recruitment domain (CARD) interactions. Based on these interactions, inflammasome sensors assemble into complexes with or without the adaptor protein ASC. However, efficient cytokine processing requires ASC recruitment and assembly into the inflammasome [17, 18].

When clustered procaspases undergo autoactivation to mature caspase-1, the inflammasome is considered active and can convert pro-IL-1β and pro-IL-18 to their mature forms [19]. Cleavage of Gasdermin D (GSDMD) by either caspase-1 or caspase-11 is critical for the induction of pyroptosis [20]. GSDMD is composed of carboxy (C) and amino (N) terminus fragments, with the C-terminus autoinhibiting the pyroptotic and inflammatory actions of the N-terminus fragment, with the latter being recruited to the cell membrane to form a pore (GSDMD-N). Caspase-11 cleaves GSDMD in response to intracellular LPS from Gram-negative bacteria [21]. Upon cleavage by caspase-11, the amino fragment of GSDMD-N initiates pyroptosis and is also associated with NLRP3 inflammasome activation, leading to upregulation of IL-1β [21]. In addition, GSDMD knockout mice are protected from lethal doses of LPS and release lower levels of IL-1β [21] (Fig. 3).

Caspase-1 also cleaves GSDMD to liberate GSDMD-N [22]. There have also been other mechanistic findings that support these studies. For instance, the GSDMD-N fragment associates with membrane lipids such as phosphatidylcholine and cholesterol, leading to pore formation [23]. The pores allow for potassium efflux, which is necessary for NLRP3 activation and release of IL-1β and IL-18, thus promoting inflammation [23].

The non-canonical pathway is activated by rodent caspase-11 and its human orthologs, caspase-4 and caspase-5 [24]. Interestingly, caspase-11 is less efficient in IL-1β and IL-18 processing than it is in GSDMD cleavage, and the caspases of the non-canonical pathway. This dual action also requires strict control to prevent pyroptosis [24]. One such control mechanism involves the transient receptor protein channel 1 (TRPC1), a non-selective ion channel, which is itself a substrate for caspase-11. Potassium efflux through pannexin-1 channels is necessary for NLRP3 inflammasome activation and pyroptosis, and TRPC1 knockouts have demonstrated stronger inflammatory responses [24]. However, the relationship between TRPC1 and GSDMD and the exact TRPC1 mechanism of action are both currently unknown (Fig. 5).

## NOD-like receptors, absent in melanoma-like receptors, and MS

Several inflammasomes have been described to contribute to the pathology of MS, thus highlighting the overlap in function among different inflammasomes [25–29]. Here, we summarize the known contribution to date of the different inflammasomes to MS.

### The NLRP3 inflammasome

**Fig. 4:**
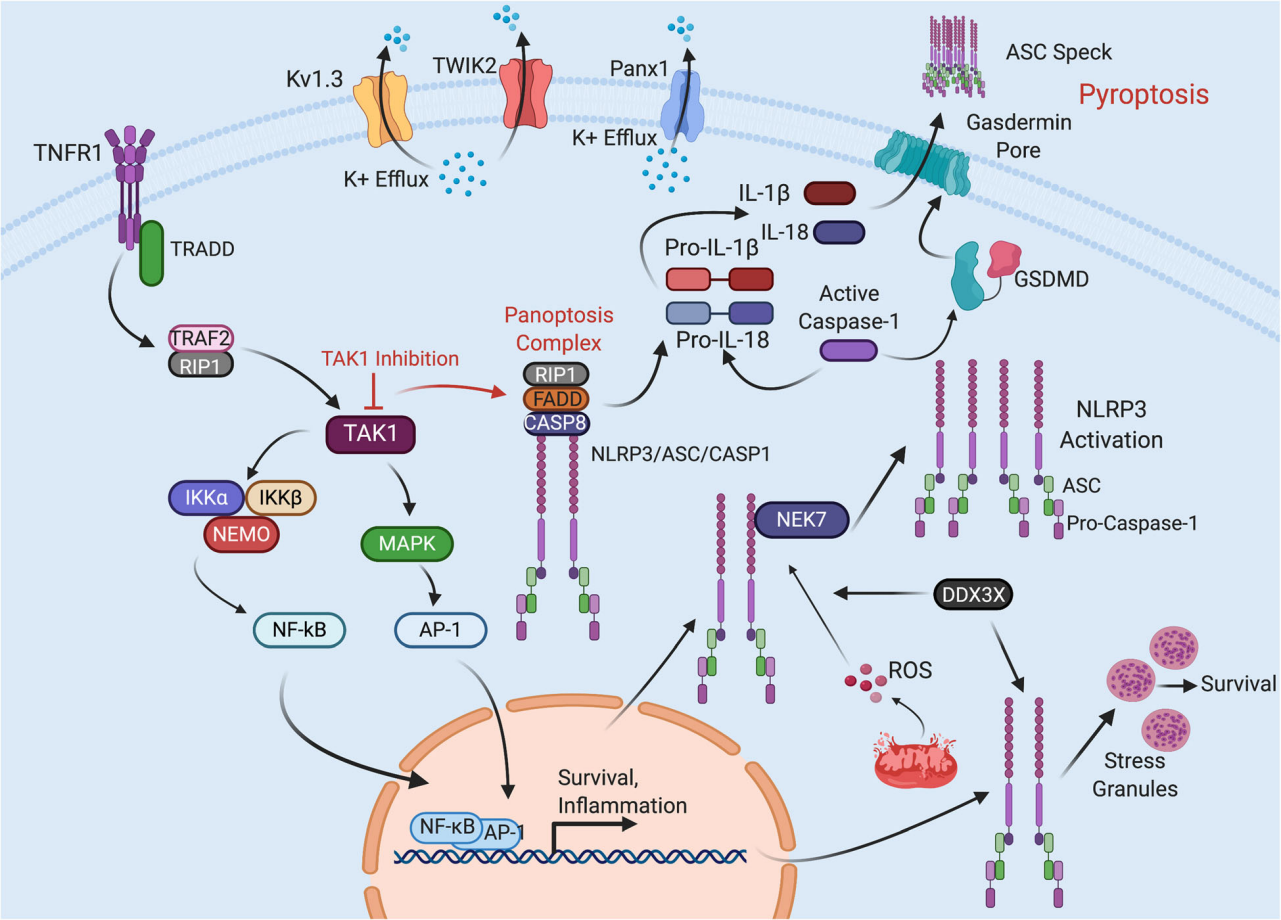
Modulation of NLRP3 signaling pathways. Upon stimulation, the tumor necrosis factor receptor 1 (TNFR1) and tumor necrosis factor receptor type 1-associated DEATH domain protein (TRADD) forms a complex in which receptor-interacting protein 1 (RIP1) acquires a polyubiquitin chain. Transforming growth factor beta-activated kinase 1 (TAK1, also known as mitogen-activated protein kinase kinase kinase 7 (MAP3K7)) binds to the polyubiquitin chain and activates the IκB kinase (IKK) complex (IKKα/IKKβ/NEMO (NF-kappa-B essential modulator)), leading to the activation of NF-*κ*B. TAK1 also activates MAP kinases (MAPK) that activate the transcription factor, activator protein 1 (AP-1). NF-κB and AP-1 induce expression of inflammatory cytokines and antiapoptotic proteins. Inhibition of TAK1 promotes the assembly of the Panoptotic cell death complex. Receptor-interacting serine/threonine-protein kinase 1 (RIPK1) in association with Fs7-associated cell surface antigen (Fas)-associated death domain (FADD) and caspase-8 (Casp8) triggers nucleotide-binding oligomerization domain-like receptor family pyrin domain-containing 3 (NLRP3) inflammasome leading to activation of caspase-1 (CASP1) and cleavage of pro-interleukin (IL)-1β and pro-IL-18 into mature cytokines and caspase-1-mediated cleavage of Gasdermin D (GSDMD) resulting in release of IL-1β, IL-18, and apoptosis speck-like staining protein containing a CARD domain (ASC) specks. DEAD-box helicase 3 X-linked (DDX3X) promotes NLRP3 inflammasome activation and the pro-death cell-fate decision by interacting with the NLRP3 NACHT domain through its helicase (Heli) domain. Induction of stress granules causes the sequestration of DDX3X making it unavailable for NLRP3 inflammasome activation and thereby leading to a pro-survival state. Serine/threonine-protein kinase NEK7 interacts with the NACHT domain of NLRP3 inducing NLRP3 activation. NLRP3 is also activated by potassium efflux mediated by two-pore domain K^+^ (TWIK2) and voltage-gated potassium channel (K_v1._3) and pannexin1 (Panx1).

The NLR family, caspase activation and recruitment domain (CARD)-containing 3 (NLRP3) sensor has been most intensely investigated of all inflammasome sensor molecules. However, currently, there is no commonly agreed upon mechanism of activation of NLRP3. Activation of NLRP3 involves two steps: priming and activation. The priming phase of NLRP3 involves activation of membrane TLRs via PAMP or DAMP binding. TLR activation (typically TLR4) signaling is transduced intracellularly to activate nuclear factor (NF)-κB, which induces the expression of NLRP3, pro-IL-1β, pro-IL-18, and pro-caspase-1. Activation of NLRP3 is associated with numerous PAMPs and DAMPs, and NLRP3 can be activated by PAMPs associated with bacteria, viruses, and fungi [30], some of which can also activate other inflammasomes. Upon activation, caspase-1 is cleaved and once active it cleaves pro-IL1β and pro-IL-18 into mature forms (IL-1β and IL-18) (Fig. 4). ASC-dependent inflammasome activation occurs through two nucleation events. First, the sensor nucleates ASC to form filaments or fibrils; then, ASC nucleates caspase-1 *via* the CARD domain to form ASC specks that amplify inflammasome activation [31].

Studies have shown that a pathogen may engage multiple inflammasome sensors within a single cell [32, 33]. Importantly, activation of multiple inflammasomes all converge to form a single supramolecular assembly and ASC speck per cell. These studies indicate that there is a cross-talk between different inflammasome sensors, but it is unclear how these inflammasome signals interplay. Moreover, ASC specks are released into tissue fluids and are phagocytosed by adjacent cells where ASC drives inflammasome nucleation in the recipient cell. Thus, ASC specks serve to perpetuate inflammasome signaling by amplifying inflammasome activation through multiple mechanisms [34]. Furthermore, it has been previously demonstrated that non-functional CARD domains or CARD only proteins block formation of ASC specks and decrease cytokine processing [35].

NLRP3 activation is tightly regulated to prevent spontaneous activation of pyroptotic and pro-inflammatory mechanisms [36]. For example, the TNF family of cytokine receptors plays important roles in inflammation and cell death and shapes the nature of the host innate and adaptive immune response. Often, the outcome of triggering these cell surface receptors is cell survival and cytokine production (Fig. 4). In immune cells, TNF binding to TNFR1 induces formation of the receptor-interacting protein 1 (RIP1)-containing complex that promotes pro-survival and inflammatory responses [37, 38]. The inactivation of transforming growth factor-activated kinase 1 (TAK1, also known as mitogen-activated protein kinase kinase kinase 7, MAP3K7) results in the assembly of the Panoptosome complex (Fig. 4). Recent studies show that this complex plays a key role in activation of the NLRP3 inflammasome and pyroptosis when TAK1 is inhibited [39–42]. DEAD-box helicase 3 X-linked (DDX3X) is another NLRP3 regulator that acts as a final checkpoint for either cell survival or pyroptosis [43]. DDX3X performs this checkpoint function by differentially associating with stress granules (cytoplasmic compartments of protein-bound mRNA) or NLRP3 to promote cell survival or pyroptosis, respectively [43]. Interestingly, DDX3X association with stress granules was specific for NLRP3 inhibition and did not affect the Absent in Melanoma 2 (AIM2) or NLR family CARD domain-containing 4 (NLRC4) [43]. In addition, the protein Never in Mitosis Gene-A (NIMA)-related kinase-7 (NEK7) is another regulator of NLRP3. NEK7 binds to the leucine rich repeat (LRR) domain of NLRP3 in response to reactive oxygen species (ROS) to allow for NLRP3 activation [22]. Interestingly, the NLRP3-NEK7 relationship is cell cycle-dependent, with the NEK7-NLRP3 forming preferentially during interphase and undergoing limited formation during mitosis [22].

However, despite the large number factors that have been previously associated with NLRP3 activation, potassium efflux is common to many of these associated factors, and is necessary for NLRP3 activation [44]. For example, Gov et al. [45] demonstrated that addition of extracellular potassium inhibited NLRP3 activation in response to *Toxoplasma gondii* infection. Several potassium channels have been implicated in NLRP3 activation. Recently, knockouts of the TWIK2 potassium channel have been shown to inhibit NLRP3 activation, suggesting that TWIK2 is a potassium efflux channel necessary for NLRP3 activation [46]. A similar study investigating the role of the T cell K_v1.3_ channel demonstrated reduced NLRP3, caspase-1, and IL-1β expression in T cells after K_v1.3_ channel blockade [47]. In addition, pannexin-1 (Panx1) channels have been implicated to mediate K^+^ efflux responsible for inflammasome activation [48].

The NLRP3 inflammasome plays a role in recruiting T cells to the CNS and subsequently priming recruited T cells once in the CNS [49]. NLRP3 action in T cells is mediated via canonical and non-canonical pathways. In addition, an alternative NLRP3 inflammasome activating pathway involving caspase-8 has been described [50]. Activation of either the non-canonical and alternative pathway leads to increase release of IL-1β. Another function of the inflammasome is to enhance the survival of autoimmune Th17 cells. Interestingly, this function was discovered in inflammasome complexes within the Th17 cells themselves instead of microglia [51].

There is substantial evidence that NLRP3 inflammasome activation in microglia plays a role in the onset and progression of MS by recruiting activated T cells to the CNS and priming them to release cytokines that exacerbate the inflammatory response (Fig. 2) [52]. With respect to activation of NLRP3 by ROS, early research in MS pathogenesis revealed that ROS production was higher in MS patients in response to protein kinase C (PKC) activation [53]. More recently, canine models of disease mimicking MS have demonstrated upregulation of genes involved in ROS generation and downregulation of antioxidant genes such as superoxide dismutase 2 (SOD2) [54]. These findings suggest that initial disturbances in ROS regulation (either acquired or genetic) may predispose individuals to developing MS by activating the NLRP3 inflammasome.

As with any pathological state associated with inflammasome activation, CSF concentrations of IL-18 and IL-1β are elevated in MS patients. Furthermore, it appears that elevated IL-1β in CSF is concomitant with depleted IL-1 receptor antagonist (IL-1Ra), an anti-inflammatory interleukin that antagonizes IL-1β at its receptor [55]. This finding suggests that there is a more complex mechanism of autoinflammatory activity involving simultaneous upregulation of pro-inflammatory interleukins and downregulation of anti-inflammatory interleukins.

### The NLRP1 inflammasome

**Fig. 5:**
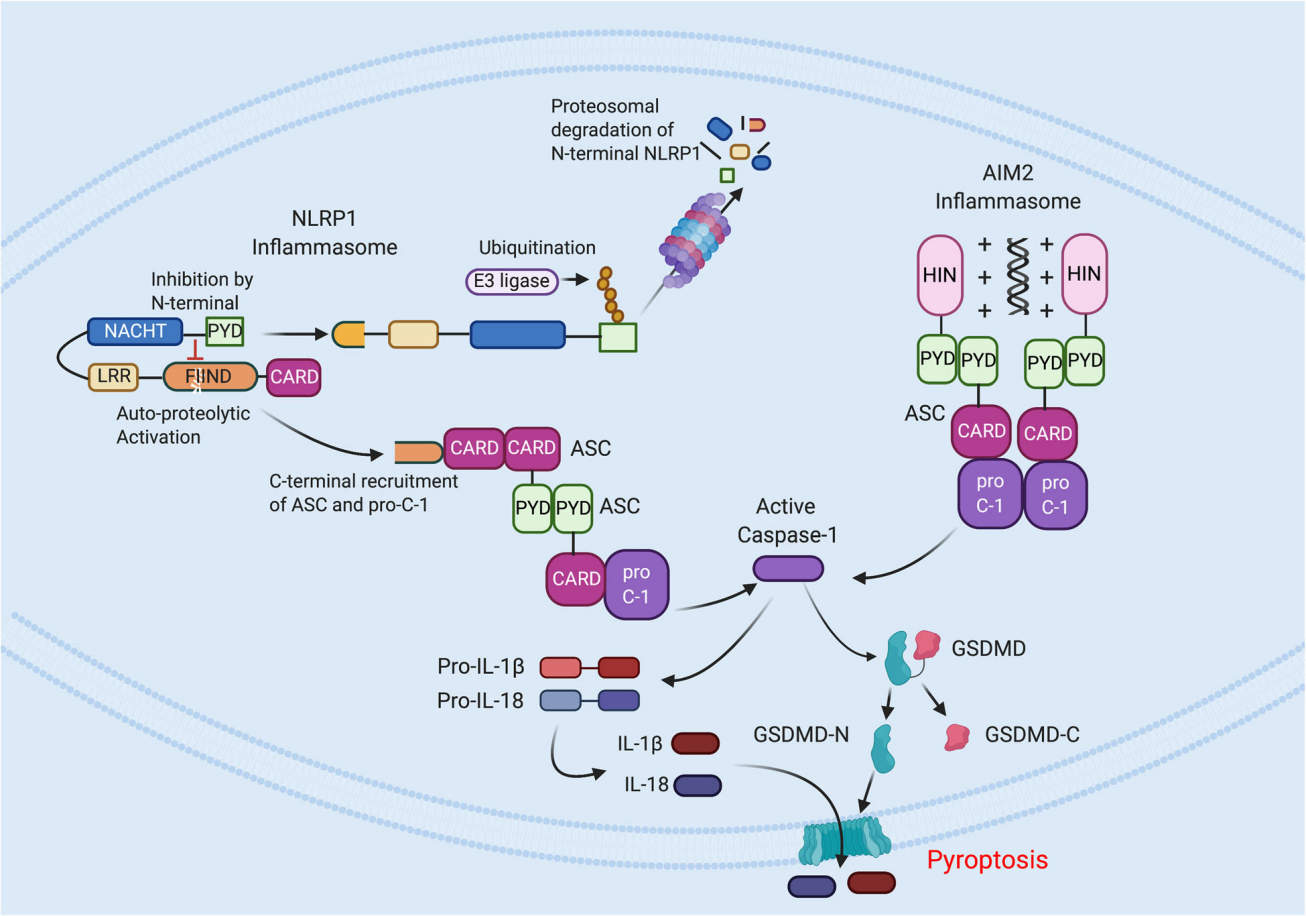
Mechanism of NLRP1 and AIM2 activation. NLR family pyrin domain-containing protein 1 (NLRP1) has a unique function to find (FIIND) domain. The amino (N)-terminus inhibits the carboxy (C) terminus effector part of the protein, which contains the caspase-recruitment domain (CARD) domain, by a non-covalent interaction. A subsequent cleavage event of the N-terminus by a pathogenic trigger results in ubiquitination of the N-terminus inhibitory fragment by ubiquitin ligases, and the latter is degraded by the proteasome. The freed effector fragment induces apoptosis speck-like protein containing a CARD domain (ASC) speck formation, followed by caspase-1 activation and in turn processing of pro-IL-1β and IL-18 as well as cleavage of GasderminD (GSDMD). Secretion of the cytokines induces inflammation and GSDMD-induced pyroptosis. Absent in melanoma 2 (AIM2) inflammasome sensing of DNA triggers recruitment of the inflammasome adaptor ASC and caspase-1. Caspase-1 directly cleaves pro-IL-1β, pro-IL-18, and GSDMD. The amino (N)-terminus GSDMD-N fragment forms pores in the plasma membrane and initiates pyroptosis. IL-1β and IL-18 are released through the GSDMD pore leading to pyroptosis. GSDMD-C, carboxy-terminus GSDMD fragment

NLRP1 is comprised of a pyrin domain in the N-terminus, a central NACHT domain, a LRR domain and a function-to-find domain (FIIND) and a CARD (Fig. 5) [56]. Thus, NLRP1 is unique with regards to its structure that in contrast to NLRP3, AIM2, pyrin, and other sensors, the C-terminus CARD of NLRP1 is part of the effector domain, which induces ASC speck formation [57, 58]. In addition, since NLRP1 has its own pyrin and CARD domain, sharing similarities to ASC, NLRP1 is capable of activating caspase-1 independently of ASC [59]. However, the precise function of the C-terminus pyrin domain, which is missing in murine NLRP1, is not clear but seems to be required for keeping NLRP1 in a self-inhibited state together with the LRRs (Fig. 5) [57]. The FIIND domain of NLRP1 has protease activity that is required for self-cleavage of NLRP1 between phenylalanine 1212 and serine 1213 [60]. F1212 and S1213 are strictly required for NLRP1 activation, in addition to histidine residue H1186. Little information is available regarding the activating ligands of NLRP1. It has been previously demonstrated that muramyl dipeptide induces NLRP1 oligomerization and ATP-binding, allowing for ASC-independent caspase-1 activation [61]. However, NLRP1 is still capable of ASC-mediated activation of caspase-1 if NLRP1 engages in autolytic cleavage of its CARD domain [62] (Fig. 5). Moreover, in the CNS, in neurons and astrocytes, high extracellular potassium has been shown to activate the NLRP1 inflammasome by a mechanism involving opening of the pannexin-1 channel, which forms protein-protein interactions with the inflammasome and with P2X7, in a process that was not found to occur in THP-1 cells [48, 63].

NLRP1 has also been associated with autoimmune diseases. For example, NLRP1 (along with NLRP3, NLRC4, and AIM2) is implicated in thyroid follicular cell death in autoimmune thyroiditis [64]. A novel autoimmune disease, NLRP1-associated autoinflammation with arthritis and dyskeratosis (NAIAD) was described in 2017 with NRLP1 as the implicated pathogenic factor [65]. However, unlike NLRP3, NLRP1 signaling is not well understood. NLRP1 variants have been reported in MS pathogenesis, but the precise mechanism of action remains undefined. A recent study identified a potentially causative amino acid substitution (glycine to serine) in NLRP1 that was associated with increased IL-18 and IL-1β production in familial MS patients [27]. However, a separate study was not able to identify pathological genetic variations in the NLRP1 gene [66]. Additional analysis of NLRP1 genetics must be performed before significant conclusions may be drawn regarding its role in familial MS.

### The NLRC4 inflammasome

NLR family, caspase activation and recruitment domain (CARD)-containing 4 (NLRC4) is another well-studied inflammasome, though there has been little research done to identify role of NLRC4 in MS. NLRC4 consists of an N-terminus CARD domain, a central nucleotide-binding domain, and a C-terminus LRR regulatory domain.

NLRC4 is similar to other inflammasomes with respect to its activation mechanism. The *Salmonella* type III secretion system and flagellin are the principal PAMPs associated with NLRC4 activation. Both of these ligands are recognized by neuronal apoptosis inhibiting proteins (NAIP), leading to NLRC4 oligomerization and caspase-1 activation [67]. With respect to MS pathology, NLRC4 is upregulated by TNF and p53 activation in response to inflammation and genotoxic events, respectively [68].

The NLRC4 inflammasome is expressed in microglia and shows altered expression in MS [69]. Primarily, NLRC4 is upregulated in the CNS during neuroinflammation, and in MS patients NLRC4 is present in regions of demyelination [70]. In a murine cuprizone model of MS, NLRC4 (in conjunction with NLRP3) was shown to induce microglial accumulation and astrogliosis, followed by demyelination. Furthermore, this sequence of microglial accumulation, astrogliosis, and demyelination was prevented in NLRC4 and NLRP3 knockouts [70]. These studies suggest a potential mechanism for CNS demyelination involving inflammasomes in glial cells.

Genetic studies investigating NLRC4 variants showed that a loss-of-function in an *NLRC4* variant (rs479333) was consistent with a beneficial response to IFN-β treatment and lower levels of IL-18 [29]. Moreover, constitutive expression of NLRP3 in MS patients could lead to disease worsening [29]. Thus, it appears that signaling through the NLRC4 and NLRP3 inflammasomes are critical regulators of the innate immune response in MS pathology.

### The AIM2 inflammasome

Very little information is available about the role of the Absent in melanoma 2 (AIM2) inflammasome in MS. AIM2 consists of an N-terminus pyrin domain and a unique C-terminus HIN domain. The pyrin domain is necessary for recruiting ASC into the complex. The HIN domain is a DNA-binding domain composed of two oligonucleotide-binding (OB) domains, and functions in detection of aberrant cytosolic DNA, such as pathogenic DNA or genomic DNA indicative of nuclear compromise (Fig. 5) [69].. In the CNS, the AIM-2 inflammasome is activated by DNA in neurons, resulting in inflammasome activation and pyroptosis [71].

AIM2 has been suggested as a drug target in the treatment of MS and is downregulated in response to IFN-β treatment in MS patients [28]. This finding is interesting, considering the dependence of type I interferon (like IFN-β) in priming the AIM2 inflammasome. One possible mechanism underlying the relationship between AIM2 and IFN-β involves GSDMD, since GSDMD suppresses the AIM2 response to cytosolic DNA and suppresses the IFN-β response [72].

## The inflammasome in the regulation of the adaptive immune response

While inflammasomes have been traditionally associated with the innate immune response, recent studies have established that inflammasomes assemble in both T and B cells [73]. Inflammasome activation was demonstrated in T and B cells in response to radiation that occurred independently of NLRP3, as NLRP3 knockout mice produced equivalent inflammatory responses as wild-type mice [74]. This study employed ionizing radiation (causing DNA damage), and therefore the AIM2 inflammasome-sensing aberrant DNA may be responsible for the increased IL-1β production noted in this investigation [75].

With respect to MS pathogenesis, ASC and NLRP3 have been detected in autoreactive Th17 cells [51, 76]. A study by Martin and colleagues found that Th17 cells constitutively release IL-1β, suggesting that Th17 cells enter the CNS and continuously promote inflammation through the release of IL-1β (Fig. 2). This idea is particularly relevant to primary and secondary progressive forms of MS. CD4 T cells form memory T cells and T_reg_ cells that suppress autoimmune activity [77]. Furthermore, T_reg_ cells suppress inflammasome activation [78]. Taken together, MS pathogenesis may involve disruption of T_reg_ cell-mediated suppression of autoimmunity. Moreover, it has been demonstrated that excess IL-1β reverses autoimmune suppression by T_reg_ cells [79].

## Inflammasome genetics and MS

While MS is not considered a true genetic disease, individuals with relatives who have been diagnosed with MS (particularly 1st degree relatives) are at increased risk of developing MS themselves (Table 1). Heritable genetic variants in key inflammasome components may predispose individuals to developing MS. Genetic variants in NLRP3 have been detected in healthy versus RRMS patients in a screen of 4 single nucleotide variants (SNV) in 150 Iranian patients and 100 controls [25]. In this study, the expression of the *NLRP3* rs3806265 C allele and CC genotype was more frequent in RRMS patients than in healthy controls, and the expression of NLRP3 was lower in patients that were in remission when compared to relapsing patients and healthy controls [25]. In contrast, Malhotra et al. screened for 14 NLRP3 SNV in 665 RRMS patients treated with IFN-β and found no association between response to IFN-β treatment and NLRP3 SNV [82]

**Table 1:** Summary of studies associating inflammasome genetics and MS

Gene	Alteration	Effect	Reference
*NLRP3*	⇒ Upregulated in RRMS patients	Increased recruitment and priming of T cells	Imani et al. [25]
	⇒ Downregulated in healthy controls	Increased serum IL-18 concentrations	Soares et al. [29]
	⇒ Gain of function mutation associated with increased severity	Degree of rarity associated with increased severity in sporadic and familial MS	Vidmar et al. [80]
	⇒ Multiple rare variants		
*NLRP1*	⇒ Multiple rare variants	Degree of rarity associated with increased severity in sporadic and familial MS	Vidmar et al. [80]
*NLRC4*	⇒ Protective intronic variants	Decreased serum IL-18 concentrations	Soares et al. [29]
*NLRP12*	⇒ Rare exomic variant	Potential disruption of pro-inflammatory network	Vilarino-Guell et al. [62]
*CASP1*	⇒ Upregulated in RRMS patients	May predict progression to RRMS from CIS	Hagman et al. [81]
	⇒ Multiple rare variants	Degree of rarity associated with increased severity in sporadic and familial MS	Vidmar et al. [80]
*PYCARD*	⇒ Upregulated in RRMS patients	May predict progression to RRMS from CIS	Hagman et al. [81]

Analysis of gene expression in RRMS patients revealed increased expression of *CASP1* (encoding caspase-1) and *PYCARD* (encoding both the PYD and CARD domains of ASC). More importantly, alterations in expression of these genes were identified in patients who progressed to RRMS after initially presenting with clinically isolated syndrome (CIS), suggesting that *PYCARD* and *CASP1* may predict progression to RRMS. Most patients presenting with CIS progress to MS, suggesting a potential mechanism underlying this progression [81].

Genetic variations in inflammasome genes also alter the severity of MS. A recent study by Soares et al. revealed that gain of function mutations in NLRP3 (Q705K) and IL-1β (-511 C > T) are associated with increased severity of MS. An intronic genetic variant in the NLRC4 gene results in reduced expression of NLRC4, serving as a protective mechanism in MS progression. This study also found that genetic variations produced a protective function due to lower serum IL-18 concentrations; namely, gain of function mutations in NLRP3 were associated with increased IL-18 concentrations while the intronic genetic variants resulted in decreased IL-18 concentrations [29]. Moreover, a loss-of-function NLRC4 variant (rs479333) was consistent with a beneficial response to IFN-β treatment and the -511 C > T SNV was consistent with increased frequency in PPMS than in RRMS [29].

Rare variants in key inflammasome genes are present in both sporadic and familial forms of MS. A recent analysis of over 1000 alleles demonstrated that *NLRP1*, *NLRP3*, and *caspase-1* variants were all significantly associated with the onset of both sporadic and familial forms of MS. Additionally, there was a significant association between variant rarity and pathogenicity, with progressively rarer variations in these three genes associated with increased MS severity [80]. Rare exome variants in NLRP12 have also been implicated in a potentially disrupted pro-inflammatory network that may predispose individuals to familial forms of MS [62].

## Inflammasome proteins as biomarkers

The ability to predict the onset of MS relapses or to assess the prognoses of MS patients requires the availability of biomarkers. CSF biomarkers are invasive and imaging biomarkers are expensive modalities that cannot be performed frequently in patients. Thus, there is a need for less invasive procedures and tests of diagnosing MS, monitoring response to treatment or monitoring diseases progression. To this extent, inflammasome proteins have been shown to be promising biomarkers of the inflammatory components in a variety of diseases and conditions such as stroke [83], depression [84], brain injury [85, 86], and MS [87].

It has been demonstrated that both ASC and caspase-1 are elevated in the serum of MS patients, suggesting that these inflammasome components are good biomarkers of MS [87]. This study evaluated the reliability of ASC and caspase-1 as biomarkers of MS and found area under the curve (AUC) values of 0.94 and 0.85 for ASC and caspase-1, respectively. For ASC, the authors found a cutoff point of 352.4 pg/mL (84% sensitivity, 90% specificity) and for caspase-1 the cutoff point was 1.30 pg/mL (89% sensitivity, 56% specificity), demonstrating the reliability of ASC and caspase-1 as biomarkers of MS [87].

A recent study identified that NLRP3 was overexpressed in the monocytes of primary progressive MS patients when compared to healthy controls. Furthermore, in this study the rate of MS progression correlated with the degree of IL-1β elevation in peripheral monocytes via RNA sequencing [88]. The utility of NLRP3 as a potential biomarker in MS is further supported by another study demonstrating elevated NLRP3 in neuromyelitis optica spectrum disorders [89]. Unfortunately, these studies did not evaluate the practical applications of their findings by providing AUC values. Thus, the reliability of IL-1β and NLRP3 as biomarkers remains to be determined.

## Inflammasomes as drug targets

**Fig. 6:**
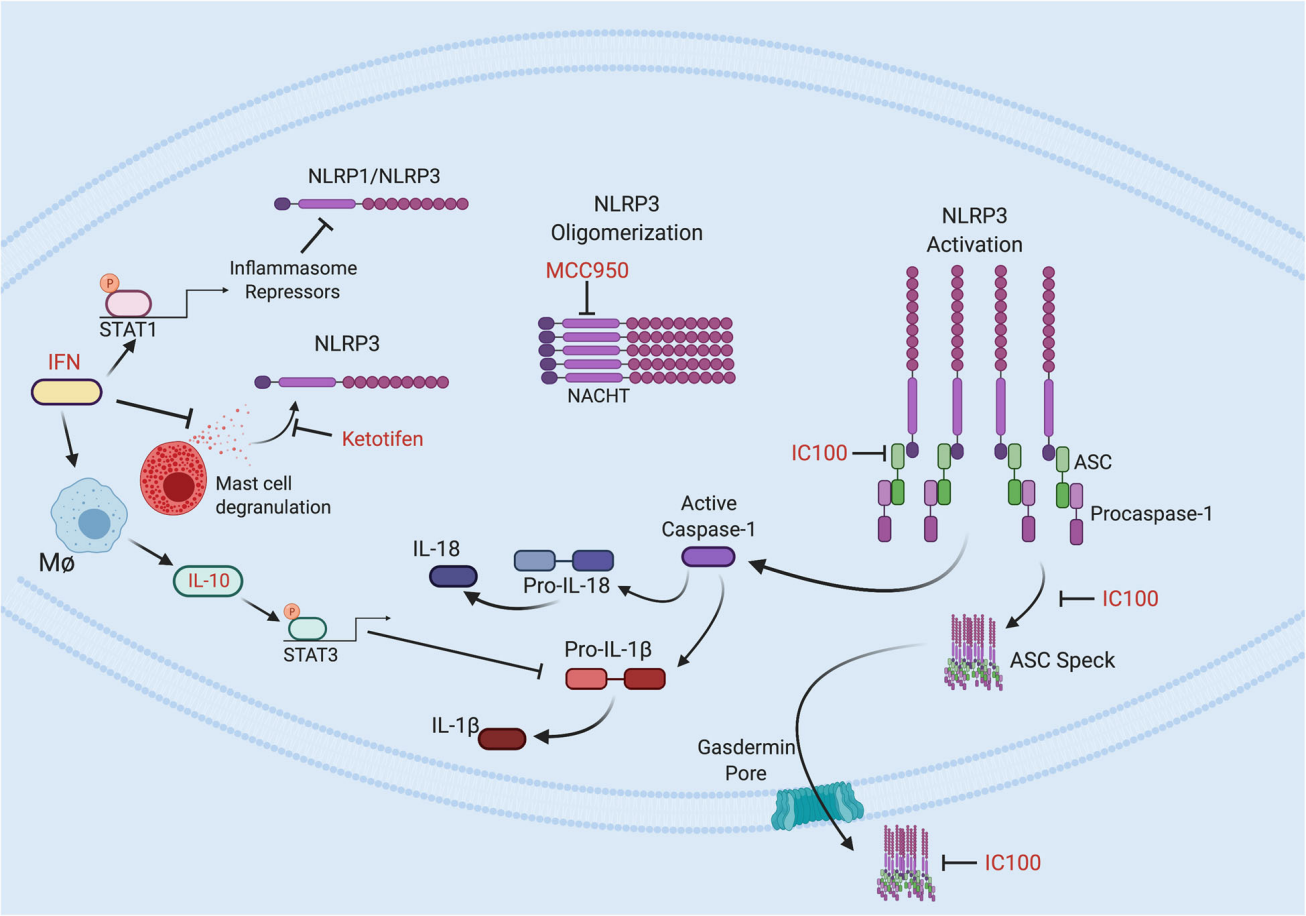
Drugs targeting inflammasome signaling in MS and EAE. Interferon (IFN) regulates the transcriptional level of signal transducer and activator transcription 1 (STAT1) to suppress activity of the (NLR) family pyrin domain-containing 1 (NLRP1) and NLRP3 inflammasomes. STAT1 target gene products directly repress these inflammasomes. Additionally, IFN/STAT1 pathway increases IL-10 synthesis in macrophages, IL-10-mediated STAT3 activation, and the suppression of interleukin (IL)-1β precursor synthesis by activated STAT3. Ketotifen is an anti-histamine that inhibits mast cell degranulation to block activation of the NLRP3 inflammasome. MCC950 directly targets the NLRP3 NACHT domain and interferes with the Walker B motif function, preventing NLRP3 conformational change and oligomerization. IC100, a humanized monoclonal antibody, binds to apoptosis speck-like staining protein containing a CARD (ASC) and prevents recruitment of ASC into the NLRP3 signaling complex and blocks formation of ASC specks intracellular and extracellularly

Inflammasome signaling pathways offer a number of possible checkpoints to develop therapeutic drugs (Fig. 6). For example, IFN-β is one of the frontline treatments of MS and may inhibit NLRP1 and NLRP3 inflammasomes [90] (Fig. 6). To gain information of the potential modulation of inflammasome activity by IFN-β, 97 patients were treated with IFN-β and classified into responders and non-responders according to clinical criteria after 24 months and clinical-radiological criteria after 12 months of treatment [91]. Baseline mRNA expression levels for NLRP3 and IL-1β were increased in peripheral blood mononuclear cells from non-responders compared to responders. These results point to a role of the NLRP3 inflammasome and its related cytokine IL1-β in the response to IFN-β in patients with RRMS. This idea is supported by the findings that IFN-β is effective in attenuating the progression of EAE [92]. It has been shown that IFN-β inhibits type I IFN signaling. IFN regulates the transcriptional level of signal transducer and activator transcription 1 (STAT1) to suppress activity of the (NLR) family pyrin domain-containing 1 (NLRP1) and NLRP3 inflammasomes [90]. On the one hand, STAT1 target gene products directly repress these inflammasomes. Alternatively, IFN/STAT1 pathway increases IL-10 synthesis in macrophages, IL-10-mediated STAT3 activation and the suppression of interleukin (IL)-1β precursor synthesis by activated STAT3 [93] (Fig. 6). However, it appears that IFN-β does not affect AIM2 activation, an interesting finding considering AIM2’s dependence on type I interferon for activation [93]. However, contradictory findings were reported in a clinical trial by Noroozi and colleagues, who found significantly decreased levels of AIM2 expression in MS patients after treatment with IFN-β [28]. Thus, more research is necessary to understand the effect of IFN-β on AIM2 signaling.

In another study of RRMS patients, it was found that IL-10 levels of those receiving IFN-β were significantly higher than control groups in the study [94]. These results support the idea that anti-inflammatory cytokine IL-10 plays a role in the clinical advantage of IFN-β in MS treatment. Lastly, Sun et al. [95] demonstrated that IL-10 inhibits IL-1β production and NLRP3 activation in microglia resulting in inhibition of caspase-1-mediated IL-1β maturation (Fig. 6).

Recently, small molecule inhibitors of NLRP3 have been developed and tested in animal models of MS. One such novel compound is MCC950. MCC950 directly targets the NLRP3 NACHT domain and interferes with the Walker B motif function thus preventing NLRP3 conformational changes and oligomerization (Fig. 6) [96]. MCC950 improved allodynia threshold in a murine model of RRMS and attenuated relapses [97]. A later study investigated the value of a rapamycin-MCC950 combination and found that MCC950 enhanced the activity of rapamycin (immunosuppressant) and that this combination was effective in mitigating MS symptoms and cytokine release [98].

IC100 is the most recently developed inflammasome inhibitor and is a humanized antibody developed against the ASC component of inflammasomes, thus having the ability to inhibit several inflammasomes including NLRP1, NLRP3, NLCR4 and AIM2. As shown in Fig. 6, ASC mediates amplification of the inflammasome response by at least three mechanisms: (1) In the presence of ASC, caspase-1 can activate a greater number of caspase-1 molecules; (2) caspase-1-mediated processing of pro-IL-1β and pro-IL-18 affects infiltration of immune cells, hence exacerbating the inflammatory response; and (3) ASC specks are released by pyroptosis and taken up by neighboring cells, promoting ASC assembly in the target cells [34]. Thus, it is possible that IC100 may interfere with inflammasome signaling at various levels of amplification. In the EAE model in mice, ASC inhibition by IC100 was demonstrated to reduce CD4 and CD8 T cell infiltration into the spinal cord and to improve functional outcomes [99]. This effect of the antibody is significant, as spinal cord infiltration is a prognostic indicator of EAE progression and severity. This novel antibody treatment was also shown to reduce microglial activation [100, 101], which as discussed previously, is a hallmark of MS pathogenesis and progression. However, it is unclear at the present time whether IC100 treatment blocks the inflammasome amplification step by interfering with ASC speck assembly in recipient cells after uptake of ASC specks. A similar approach inhibiting the inflammasome using an antibody against ASC has been successfully used after traumatic brain injury [102] and spinal cord injury [103] both of which produce neuronal loss and demyelination associated with secondary injury, yet inflammasome inhibition by neutralizing ASC resulted in improved histopathological and functional outcomes.

Ketotifen, an anti-histamine, has been demonstrated to have a potential application in MS [104]. In an EAE model, the protective effect was concomitant with decreased NLRP3 inflammasome activation, rebalanced oxidative stress and reduced T cell infiltration in the CNS. Even though ketotifen administration does not seem to alter mast cell infiltration in the CNS, it decreased enzymes typically produced by these cells. However, a caveat to this study is that ketotifen was only able to attenuate EAE progression if administered early, defined by the authors as within 7–17 days post-induction of EAE [104].

## Conclusion

There is ample evidence that activation of inflammasome signaling pathways plays a crucial role in MS pathogenesis and progression. Genetic deficiencies in inflammasome regulatory genes are associated with both MS cases and EAE models. As reviewed in this report, elevated levels of inflammasome signaling proteins (NLRP3, ASC, caspase-1) and pro-inflammatory cytokines (IL-1β and IL-18) are elevated in serum of MS patients and in the spinal cord of EAE animals. However, most studies have largely focused on the role of NLRP3 in MS, but further information is needed about the regulation of other inflammasome pathways in MS pathogenesis. For example, there is a lack of knowledge regarding whether targeted inflammasome inhibition through drug therapy silences inflammatory events regulated by one inflammasome or whether it dampens a multitude of inflammasome signaling pathways upregulated in MS. In addition, little information is available as to the role of ASC specks in MS pathogenesis and a better understanding of the role of immune cells that present active inflammasomes in demyelination of the spinal cord. Lastly, it is not clear how inflammasome signaling affects the Th1 and Th17 immune responses in MS or how the inflammasome is differentially regulated on RRMS, PPMS and SPMS. Taken together, the inflammasome plays a crucial role in MS pathogenesis. Development of therapeutics that target inflammasome signaling pathways may dampen damaging inflammatory events and result in improve functional outcomes.

## Data Availability

N/A

## Abbreviations

AIM2: Absent in melanoma 2
ALR: Aim2-like receptor
APC: Antigen presenting cell
ASC: Apoptosis-associated speck-like protein containing a caspase-recruitment domain
AUC: Area under the curve
BBB: Blood-brain barrier
C: Carboxy
CARD: Caspase-recruitment domain
Casp8: Caspase-80
CNS: Central nervous system
CSF: Cerebrospinal fluid
CIS: Clinically isolated syndrome
DAMP: Danger associate molecular patterns
DC: Dendritic cells
DDX3X: DEAD-box helicase 3 X-linked
FADD: Fas-associated death domain
FIIND: Function-to-find domain
GM-CSF: Granulocyte-macrophage colony stimulating factor
GSDMD: Gasdermin D
GSDMD-C: Carboxy-terminus domain of GSDMD
GSDMD-N: Amino-terminus domain of GSDMD
HIN: Hematopoietic interferon-inducible nuclear protein
IFN: Interferon
IKK: IκB kinase
IL: Interleukin
IL-1Ra: IL-1 receptor antagonist
LRR: Leucine rich repeat
IκB: Inhibitor NFκB
MS: Multiple sclerosis
NACHT: NAIP (neuronal apoptosis inhibitory protein), CIITA (MHC class II transcription activator), HET-E (incompatibility locus protein from *Podospora anserina*), and TP1 (telomerase-associated protein) domain
MAP3K7: Mitogen-activated protein kinase kinase kinase 7
Mt DNA: Mitochondrial deoxyribonucleic acid
MYD88: Myeloid differentiation primary response 88
N: Amino
NAIP: Neuronal apoptosis inhibiting protein
NAIAD: NLRP1-Associated Autoinflammation with Arthritis and Dyskeratosis
NIMA: Never in Mitosis Gene-A
NLRC4: NLR family, caspase activation and recruitment domain (CARD)-containing 4
NFκB: Nuclear factor-κB
NLR: NOD-like receptor
NLRP3: NLR family pyrin domain-containing protein 1
PAMP: Pathogen-associated molecular pattern
Panx1: Pannexin 1
PRR: Pattern recognition receptors
PPMS: Primary progressive multiple sclerosis
PKC: Protein kinase C
PYD: Pyrin
RIP1: Receptor-interacting protein
ROS: Reactive oxygen species
RRMS: Relapsing-remitting multiple sclerosis
SPMS: Secondary progressive multiple sclerosis
SOD2: Superoxide dismutase 2
STAT1: Signal transducer and activator transcription 1
STAT3: Signal transducer and activator transcription 3
TLR: Toll-like receptor
T_eff_: T effector
T_reg_: T regulatory
TRADD: TNFR1-associated DEATH domain protein
TRPC1: Transient receptor protein channel 1
TNF: Tumor necrosis factor
TNFR1: Tumor necrosis factor receptor 1
TWIK2: Two-pore domain K^+^

## References

[1] McGinley AM, Edwards SC, Raverdeau M, Mills KHG. Th17cells, gammadelta T cells and their interplay in EAE and multiple sclerosis. J Autoimmun. 2018;21;S0896-8411(18)30007-6. 10.1016/j.jaut.2018.01.001 29395738

[2] Villar LM, Sadaba MC, Roldan E, Masjuan J, Gonzalez-Porque P, Villarrubia N, Espino M, Garcia-Trujillo JA, Bootello A, Alvarez-Cermeno JC (2005). Intrathecal synthesis of oligoclonal IgM against myelin lipids predicts an aggressive disease course in MS. J Clin Invest.

[3] Kinnunen T, Chamberlain N, Morbach H, Cantaert T, Lynch M, Preston-Hurlburt P, Herold KC, Hafler DA, O'Connor KC, Meffre E (2013). Specific peripheral B cell tolerance defects in patients with multiple sclerosis. J Clin Invest.

[4] Li R, Patterson KR, Bar-Or A (2018). Reassessing B cell contributions in multiple sclerosis. Nat Immunol.

[5] Jelcic I, Al Nimer F, Wang J, Lentsch V, Planas R, Jelcic I, Madjovski A, Ruhrmann S, Faigle W, Frauenknecht K (2018). Memory B cells activate brain-homing, autoreactive CD4(+) T cells in multiple sclerosis. Cell.

[6] Garg N, Smith TW (2015). An update on immunopathogenesis, diagnosis, and treatment of multiple sclerosis. Brain Behav.

[7] Markowitz CE (2013). Multiple sclerosis update. Am J Manag Care.

[8] Katz Sand I (2015). Classification, diagnosis, and differential diagnosis of multiple sclerosis. Curr Opin Neurol.

[9] Wilhelm H, Schabet M (2015). The diagnosis and treatment of optic neuritis. Dtsch Arztebl Int.

[10] Correale J, Gaitán MI, Ysrraelit MC, Fiol MP (2017). Progressive multiple sclerosis: from pathogenic mechanisms to treatment. Brain.

[11] Latz E, Xiao TS, Stutz A (2013). Activation and regulation of the inflammasomes. Nature reviews Immunology.

[12] Miao EA, Rajan JV, Aderem A (2011). Caspase-1-induced pyroptotic cell death. Immunological reviews.

[13] Takeuchi O, Akira S (2010). Pattern recognition receptors and inflammation. Cell.

[14] Vajjhala PR, Mirams RE, Hill JM (2012). Multiple binding sites on the pyrin domain of ASC protein allow self-association and interaction with NLRP3 protein. J Biol Chem.

[15] Zhou R, Yazdi AS, Menu P, Tschopp J (2011). A role for mitochondria in NLRP3 inflammasome activation. Nature.

[16] Misawa T, Takahama M, Kozaki T, Lee H, Zou J, Saitoh T, Akira S (2013). Microtubule-driven spatial arrangement of mitochondria promotes activation of the NLRP3 inflammasome. Nat Immunol.

[17] Mariathasan S, Newton K, Monack DM, Vucic D, French DM, Lee WP, Roose-Girma M, Erickson S, Dixit VM (2004). Differential activation of the inflammasome by caspase-1 adaptors ASC and Ipaf. Nature.

[18] Van Opdenbosch N, Gurung P, Vande Walle L, Fossoul A, Kanneganti TD, Lamkanfi M (2014). Activation of the NLRP1b inflammasome independently of ASC-mediated caspase-1 autoproteolysis and speck formation. Nat Commun.

[19] Martinon F, Burns K, Tschopp J (2002). The inflammasome: a molecular platform triggering activation of inflammatory caspases and processing of proIL-β. Mol Cell.

[20] Kesavardhana S, Malireddi RKS, Kanneganti TD (2020). Caspases in cell death, inflammation, and pyroptosis. Annu Rev Immunol.

[21] Kayagaki N, Stowe IB, Lee BL, O'Rourke K, Anderson K, Warming S, Cuellar T, Haley B, Roose-Girma M, Phung QT (2015). Caspase-11 cleaves gasdermin D for non-canonical inflammasome signalling. Nature.

[22] Shi H, Wang Y, Li X, Zhan X, Tang M, Fina M, Su L, Pratt D, Bu CH, Hildebrand S (2016). NLRP3 activation and mitosis are mutually exclusive events coordinated by NEK7, a new inflammasome component. Nat Immunol.

[23] Aglietti RA, Estevez A, Gupta A, Ramirez MG, Liu PS, Kayagaki N, Ciferri C, Dixit VM, Dueber EC (2016). GsdmD p30 elicited by caspase-11 during pyroptosis forms pores in membranes. Proc Natl Acad Sci U S A.

[24] Matikainen S, Nyman TA, Cypryk W (2020). Function and regulation of noncanonical caspase-4/5/11 inflammasome. J Immunol.

[25] Imani D, Azimi A, Salehi Z, Rezaei N, Emamnejad R, Sadr M, Izad M (2018). Association of nod-like receptor protein-3 single nucleotide gene polymorphisms and expression with the susceptibility to relapsing-remitting multiple sclerosis. Int J Immunogenet.

[26] Inoue M (2015). Shinohara ML: [NLRP3 inflammasome and multiple sclerosis/EAE]. Nihon Rinsho.

[27] Maver A, Lavtar P, Ristić S, Stopinšek S, Simčič S, Hočevar K, Sepčić J, Drulović J, Pekmezović T, Novaković I (2017). Identification of rare genetic variation of NLRP1 gene in familial multiple sclerosis. Sci Rep.

[28] Noroozi S, Meimand HAE, Arababadi MK, Nakhaee N, Asadikaram G (2017). The effects of IFN-β 1a on the expression of inflammasomes and apoptosis-associated speck-like proteins in multiple sclerosis patients. Mol Neurobiol.

[29] Soares JL, Oliveira EM, Pontillo A (2019). Variants in NLRP3 and NLRC4 inflammasome associate with susceptibility and severity of multiple sclerosis. Mult Scler Relat Disord.

[30] Swanson KV, Deng M, Ting JPY (2019). The NLRP3 inflammasome: molecular activation and regulation to therapeutics. Nature Reviews Immunology.

[31] Lu A, Magupalli VG, Ruan J, Yin Q, Atianand MK, Vos MR, Schroder GF, Fitzgerald KA, Wu H, Egelman EH (2014). Unified polymerization mechanism for the assembly of ASC-dependent inflammasomes. Cell.

[32] Broz P, Newton K, Lamkanfi M, Mariathasan S, Dixit VM, Monack DM (2010). Redundant roles for inflammasome receptors NLRP3 and NLRC4 in host defense against Salmonella. J Exp Med.

[33] Karki R, Man SM, Malireddi RKS, Gurung P, Vogel P, Lamkanfi M, Kanneganti TD (2015). Concerted activation of the AIM2 and NLRP3 inflammasomes orchestrates host protection against Aspergillus infection. Cell Host Microbe.

[34] Sharma D, Kanneganti TD (2016). The cell biology of inflammasomes: mechanisms of inflammasome activation and regulation. J Cell Biol.

[35] Matusiak M, Van Opdenbosch N, Lamkanfi M (2015). CARD- and pyrin-only proteins regulating inflammasome activation and immunity. Immunol Rev.

[36] Kesavardhana S, RKS M, Kanneganti T-D (2020). Caspases in cell death, inflammation, and pyroptosis.

[37] Peltzer N, Darding M, Walczak H (2016). Holding RIPK1 on the ubiquitin leash in TNFR1 signaling. Trends Cell Biol.

[38] Ting AT, Bertrand MJM (2016). More to life than NF-kappaB in TNFR1 signaling. Trends Immunol.

[39] Malireddi RKS, Gurung P, Kesavardhana S, Samir P, Burton A, Mummareddy H, Vogel P, Pelletier S, Burgula S, Kanneganti TD. Innate immune priming in the absence of TAK1 drives RIPK1 kinase activity-independent pyroptosis, apoptosis, necroptosis, and inflammatory disease. J Exp Med. 2020;217(3):e20191644. 10.1084/jem.20191644 PMC7062518 31869420

[40] Malireddi RKS, Gurung P, Mavuluri J, Dasari TK, Klco JM, Chi H, Kanneganti T-D (2018). TAK1 restricts spontaneous NLRP3 activation and cell death to control myeloid proliferation. J Exp Med.

[41] Orning P, Weng D, Starheim K, Ratner D, Best Z, Lee B, Brooks A, Xia S, Wu H, Kelliher MA (2018). Pathogen blockade of TAK1 triggers caspase-8-dependent cleavage of gasdermin D and cell death. Science.

[42] Sarhan J, Liu BC, Muendlein HI, Li P, Nilson R, Tang AY, Rongvaux A, Bunnell SC, Shao F, Green DR, Poltorak A (2018). Caspase-8 induces cleavage of gasdermin D to elicit pyroptosis during Yersinia infection. Proc Natl Acad Sci U S A.

[43] Samir P, Kesavardhana S, Patmore DM, Gingras S, Malireddi RKS, Karki R, Guy CS, Briard B, Place DE, Bhattacharya A (2019). DDX3X acts as a live-or-die checkpoint in stressed cells by regulating NLRP3 inflammasome. Nature.

[44] Muñoz-Planillo R, Kuffa P, Martínez-Colón G, Smith BL, Rajendiran TM, Núñez G (2013). K^+^ efflux is the common trigger of NLRP3 inflammasome activation by bacterial toxins and particulate matter. Immunity.

[45] Gov L, Schneider CA, Lima TS, Pandori W, Lodoen MB (2017). NLRP3 and potassium efflux drive rapid IL-1β release from primary human monocytes during *Toxoplasma gondii* infection. J Immunol.

[46] Di A, Xiong S, Ye Z, Malireddi RKS, Kometani S, Zhong M, Mittal M, Hong Z, Kanneganti T-D, Rehman J, Malik AB (2018). The TWIK2 potassium efflux channel in macrophages mediates NLRP3 inflammasome-induced inflammation. Immunity.

[47] Zhu J, Yang Y, Hu SG, Zhang QB, Yu J, Zhang YM (2017). T-lymphocyte K(v)1.3 channel activation triggers the NLRP3 inflammasome signaling pathway in hypertensive patients. Exp Ther Med.

[48] Silverman WR, de Rivero Vaccari JP, Locovei S, Qiu F, Carlsson SK, Scemes E, Keane RW, Dahl G (2009). The pannexin 1 channel activates the inflammasome in neurons and astrocytes. J Biol Chem.

[49] Inoue M, Williams KL, Gunn MD, Shinohara ML (2012). NLRP3 inflammasome induces chemotactic immune cell migration to the CNS in experimental autoimmune encephalomyelitis. Proc Natl Acad Sci U S A.

[50] Antonopoulos C, Russo HM, El Sanadi C, Martin BN, Li X, Kaiser WJ, Mocarski ES, Dubyak GR (2015). Caspase-8 as an effector and regulator of NLRP3 inflammasome signaling. J Biol Chem.

[51] Martin BN, Wang C, Zhang CJ, Kang Z, Gulen MF, Zepp JA, Zhao J, Bian G, Do JS, Min B (2016). T cell-intrinsic ASC critically promotes T(H)17-mediated experimental autoimmune encephalomyelitis. Nat Immunol.

[52] Olcum M, Tastan B, Kiser C, Genc S, Genc K (2020). Microglial NLRP3 inflammasome activation in multiple sclerosis. Adv Protein Chem Struct Biol.

[53] Vladimirova O, Lu FM, Shawver L, Kalman B (1999). The activation of protein kinase C induces higher production of reactive oxygen species by mononuclear cells in patients with multiple sclerosis than in controls. Inflamm Res.

[54] Attig F, Spitzbarth I, Kalkuhl A, Deschl U, Puff C, Baumgärtner W, Ulrich R (2019). Reactive oxygen species are key mediators of demyelination in canine distemper leukoencephalitis but not in Theiler's murine encephalomyelitis. Int J Mol Sci.

[55] de Jong BA, Huizinga TWJ, Bollen ELEM, Uitdehaag BMJ, Bosma GPT, van Buchem MA, Remarque EJ, Burgmans ACS, Kalkers NF, Polman CH, Westendorp RGJ (2002). Production of IL-1β and IL-1Ra as risk factors for susceptibility and progression of relapse-onset multiple sclerosis. J Neuroimmunol.

[56] Tupik JD, Nagai-Singer MA, Allen IC. To protect or adversely affect? The dichotomous role of the NLRP1 inflammasome in human disease. Mol Asp Med. 2020:100858. 10.1016/j.mam.2020.100858 32359693

[57] Frew BC, Joag VR, Mogridge J (2012). Proteolytic processing of Nlrp1b is required for inflammasome activity. PLoS Pathog.

[58] Lacey CA, Miao EA (2019). NLRP1 - One NLR to guard them all. Embo J.

[59] Reubold TF, Hahne G, Wohlgemuth S, Eschenburg S (2014). Crystal structure of the leucine-rich repeat domain of the NOD-like receptor NLRP1: Implications for binding of muramyl dipeptide. FEBS Letters.

[60] Finger JN, Lich JD, Dare LC, Cook MN, Brown KK, Duraiswami C, Bertin J, Gough PJ (2012). Autolytic proteolysis within the function to find domain (FIIND) is required for NLRP1 inflammasome activity. J Biol Chem.

[61] Faustin B, Lartigue L, Bruey JM, Luciano F, Sergienko E, Bailly-Maitre B, Volkmann N, Hanein D, Rouiller I, Reed JC (2007). Reconstituted NALP1 inflammasome reveals two-step mechanism of caspase-1 activation. Mol Cell.

[62] Vilarino-Guell C, Zimprich A, Martinelli-Boneschi F, Herculano B, Wang Z, Matesanz F, Urcelay E, Vandenbroeck K, Leyva L, Gris D (2019). Exome sequencing in multiple sclerosis families identifies 12 candidate genes and nominates biological pathways for the genesis of disease. PLoS Genet.

[63] de Rivero Vaccari JP, Dietrich WD, Keane RW (2014). Activation and regulation of cellular inflammasomes: gaps in our knowledge for central nervous system injury. J Cereb Blood Flow Metab.

[64] Guo Q, Wu Y, Hou Y, Liu Y, Liu T, Zhang H, Fan C, Guan H, Li Y, Shan Z, Teng W (2018). Cytokine secretion and pyroptosis of thyroid follicular cells mediated by enhanced NLRP3, NLRP1, NLRC4, and AIM2 inflammasomes are associated with autoimmune thyroiditis. Front Immunol.

[65] Grandemange S, Sanchez E, Louis-Plence P, Tran Mau-Them F, Bessis D, Coubes C, Frouin E, Seyger M, Girard M, Puechberty J (2017). A new autoinflammatory and autoimmune syndrome associated with NLRP1 mutations: NAIAD (NLRP1-associated autoinflammation with arthritis and dyskeratosis). Ann Rheum Dis.

[66] Bernales CQ, Encarnacion M, Criscuoli MG, Yee IM, Traboulsee AL, Sadovnick AD, Vilariño-Güell C (2018). Analysis of NOD-like receptor NLRP1 in multiple sclerosis families. Immunogenetics.

[67] Bauer R, Rauch I. The NAIP/NLRC4 inflammasome in infection and pathology. Mol Asp Med. 2020:100863. 10.1016/j.mam.2020.100863 32499055

[68] Duncan JA, Canna SW (2018). The NLRC4 inflammasome. Immunol Rev.

[69] Shaw N, Liu ZJ (2014). Role of the HIN domain in regulation of innate immune responses. Mol Cell Biol.

[70] Freeman L, Guo H, David CN, Brickey WJ, Jha S, Ting JPY (2017). NLR members NLRC4 and NLRP3 mediate sterile inflammasome activation in microglia and astrocytes. J Exp Med.

[71] Adamczak SE, de Rivero Vaccari JP, Dale G, Brand FJ, Nonner D, Bullock MR, Dahl GP, Dietrich WD, Keane RW (2014). Pyroptotic neuronal cell death mediated by the AIM2 inflammasome. J Cereb Blood Flow Metab.

[72] Banerjee I, Behl B, Mendonca M, Shrivastava G, Russo AJ, Menoret A, Ghosh A, Vella AT, Vanaja SK, Sarkar SN (2018). Gasdermin D destrains type I interferon response to cytosolic DNA by disrupting ionic homeostasis. Immunity.

[73] Evavold CL, Kagan JC (2018). How inflammasomes inform adaptive immunity. J Mol Biol.

[74] Stoecklein VM, Osuka A, Ishikawa S, Lederer MR, Wanke-Jellinek L, Lederer JA (2015). Radiation exposure induces inflammasome pathway activation in immune cells. J immunol.

[75] Hu B, Jin C, Li H-B, Tong J, Ouyang X, Cetinbas NM, Zhu S, Strowig T, Lam FC, Zhao C (2016). The DNA-sensing AIM2 inflammasome controls radiation-induced cell death and tissue injury. Science (New York, NY).

[76] Arbore G, West EE, Spolski R, Robertson AAB, Klos A, Rheinheimer C, Dutow P, Woodruff TM, Yu ZX, O'Neill LA (2016). T helper 1 immunity requires complement-driven NLRP3 inflammasome activity in CD4^+^ T cells. Science.

[77] Laidlaw BJ, Craft JE, Kaech SM (2016). The multifaceted role of CD4(+) T cells in CD8(+) T cell memory. Nat Rev Immunol.

[78] Yao Y, Vent-Schmidt J, McGeough MD, Wong M, Hoffman HM, Steiner TS, Levings MK (2015). Tr1 cells, but not Foxp3+ regulatory T cells, suppress NLRP3 inflammasome activation via an IL-10-dependent mechanism. J Immunol.

[79] O'Sullivan BJ, Thomas HE, Pai S, Santamaria P, Iwakura Y, Steptoe RJ, Kay TW, Thomas R (2006). IL-1 beta breaks tolerance through expansion of CD25+ effector T cells. J Immunol.

[80] Vidmar L, Maver A, Drulovic J, Sepcic J, Novakovic I, Ristic S, Sega S, Peterlin B (2019). Multiple Sclerosis patients carry an increased burden of exceedingly rare genetic variants in the inflammasome regulatory genes. Sci Rep.

[81] Hagman S, Kolasa M, Basnyat P, Helminen M, Kahonen M, Dastidar P, Lehtimaki T, Elovaara I (2015). Analysis of apoptosis-related genes in patients with clinically isolated syndrome and their association with conversion to multiple sclerosis. J Neuroimmunol.

[82] Malhotra S, Sorosina M, Rio J, Peroni S, Midaglia L, Villar LM, Alvarez-Cermeno JC, Schroeder I, Esposito F, Clarelli F (2018). NLRP3 polymorphisms and response to interferon-beta in multiple sclerosis patients. Mult Scler.

[83] Kerr N, García-Contreras M, Abbassi S, Mejias NH, Desousa BR, Ricordi C, Dietrich WD, Keane RW, de Rivero Vaccari JP (2018). Inflammasome proteins in serum and serum-derived extracellular vesicles as biomarkers of stroke. Front Mol Neurosci.

[84] Syed SA, Beurel E, Loewenstein DA, Lowell JA, Craighead WE, Dunlop BW, Mayberg HS, Dhabhar F, Dietrich WD, Keane RW (2018). Defective inflammatory pathways in never-treated depressed patients are associated with poor treatment response. Neuron.

[85] Adamczak S, Dale G, de Rivero Vaccari JP, Bullock MR, Dietrich WD, Keane RW (2012). Inflammasome proteins in cerebrospinal fluid of brain-injured patients as biomarkers of functional outcome: clinical article. J Neurosurg.

[86] Kerr N, Lee SW, Perez-Barcena J, Crespi C, Ibañez J, Bullock MR, Dietrich WD, Keane RW, de Rivero Vaccari JP (2018). Inflammasome proteins as biomarkers of traumatic brain injury. PLoS One.

[87] Keane RW, Dietrich WD, de Rivero Vaccari JP (2018). Inflammasome proteins as biomarkers of multiple sclerosis. Front Neurol.

[88] Malhotra S, Costa C, Eixarch H, Keller CW, Amman L, Martinez-Banaclocha H, Midaglia L, Sarro E, Machin-Diaz I, Villar LM (2020). NLRP3 inflammasome as prognostic factor and therapeutic target in primary progressive multiple sclerosis patients. Brain.

[89] Peng Y, Chen J, Dai Y, Jiang Y, Qiu W, Gu Y, Wang H (2019). NLRP3 level in cerebrospinal fluid of patients with neuromyelitis optica spectrum disorders: Increased levels and association with disease severity. Mult Scler Relat Disord.

[90] Tschopp J, Schroder K (2010). NLRP3 inflammasome activation: the convergence of multiple signalling pathways on ROS production?. Nat Rev Immunol.

[91] Malhotra S, Rio J, Urcelay E, Nurtdinov R, Bustamante MF, Fernandez O, Oliver B, Zettl U, Brassat D, Killestein J (2015). NLRP3 inflammasome is associated with the response to IFN-beta in patients with multiple sclerosis. Brain.

[92] Inoue M, Shinohara ML (2013). The role of interferon-beta in the treatment of multiple sclerosis and experimental autoimmune encephalomyelitis - in the perspective of inflammasomes. Immunology.

[93] Guarda G, Braun M, Staehli F, Tardivel A, Mattmann C, Förster I, Farlik M, Decker T, Du Pasquier RA, Romero P, Tschopp J (2011). Type I interferon inhibits interleukin-1 production and inflammasome activation. Immunity.

[94] Ersoy E, Kus CN, Sener U, Coker I, Zorlu Y (2005). The effects of interferon-beta on interleukin-10 in multiple sclerosis patients. Eur J Neurol.

[95] Sun Y, Ma J, Li D, Li P, Zhou X, Li Y, He Z, Qin L, Liang L, Luo X (2019). Interleukin-10 inhibits interleukin-1beta production and inflammasome activation of microglia in epileptic seizures. J Neuroinflammation.

[96] Coll RC, Hill JR, Day CJ, Zamoshnikova A, Boucher D, Massey NL, Chitty JL, Fraser JA, Jennings MP, Robertson AAB, Schroder K (2019). MCC950 directly targets the NLRP3 ATP-hydrolysis motif for inflammasome inhibition. Nat Chem Biol.

[97] Khan N, Kuo A, Brockman DA, Cooper MA, Smith MT (2018). Pharmacological inhibition of the NLRP3 inflammasome as a potential target for multiple sclerosis induced central neuropathic pain. Inflammopharmacology.

[98] Xu L, Zhang C, Jiang N, He D, Bai Y, Xin Y (2019). Rapamycin combined with MCC950 to treat multiple sclerosis in experimental autoimmune encephalomyelitis. J Cell Biochem.

[99] Desu HL, Plastini M, Illiano P, Bramlett HM, Dietrich WD, de Rivero Vaccari JP, Brambilla R, Keane RW (2020). IC100: a novel anti-ASC monoclonal antibody improves functional outcomes in an animal model of multiple sclerosis. J Neuroinflammation.

[100] Lee SW, de Rivero Vaccari JP, Truettner JS, Dietrich WD, Keane RW (2019). The role of microglial inflammasome activation in pyroptotic cell death following penetrating traumatic brain injury. J Neuroinflammation.

[101] Lee SW, Gajavelli S, Spurlock MS, Andreoni C, de Rivero Vaccari JP, Bullock MR, Keane RW, Dietrich WD (2018). Microglial inflammasome activation in penetrating ballistic-like brain injury. J Neurotrauma.

[102] de Rivero Vaccari JP, Lotocki G, Bramlett HM, Dietrich WD, Keane RW, Alonso OF (2009). Therapeutic neutralization of the NLRP1 inflammasome reduces the innate immune response and improves histopathology after traumatic brain injury. J Cereb Blood Flow Metab.

[103] de Rivero Vaccari JP, Lotocki G, Marcillo AE, Dietrich WD, Keane RW (2008). A molecular platform in neurons regulates inflammation after spinal cord injury. J Neurosci.

[104] Pinke KH, Zorzella-Pezavento SFG, de Campos Fraga-Silva TF, Mimura LAN, de Oliveira LRC, Ishikawa LLW, Fernandes AAH, Lara VS, Sartori A (2020). Calming down mast cells with ketotifen: a potential strategy for multiple sclerosis therapy?. Neurotherapeutics.

